# Seeking Certainty? Judicial Approaches to the (Non-)Treatment of Minimally Conscious Patients

**DOI:** 10.1093/medlaw/fwx014

**Published:** 2017-04-18

**Authors:** Richard Huxtable, Giles Birchley

**Affiliations:** 1 Centre for Ethics in Medicine, School of Social and Community Medicine, University of Bristol, UK

**Keywords:** Minimally conscious state, Best interests, Balancing exercise

## Abstract

A modest, but growing, body of case law is developing around the (non-)treatment of patients in the minimally conscious state. We sought to explore the approaches that the courts take to these decisions. Using the results of a qualitative analysis, we identify five key features of the rulings to date. First, the judges appear keen to frame the cases in such a way that these are rightly matters for judicial determination. Secondly, the judges appraise the types and forms of expertise that enter the courtroom, seeming to prefer the ‘objective’ and ‘scientific’, and particularly the views of the doctors. Thirdly, the judges appear alert to the reasonableness of the evidence (and, indeed, the parties) and will look favourably on parties who are willing to co-operate. But the judges will not simply endorse any consensus reached by the parties; rather, the judges will reach their own decisions. Those decisions must be taken in the best interests of the patient. Fourthly, the judges approach this assessment in different ways. A balancing exercise is not consistently undertaken and, even in those cases in which it is, the weight accorded to particular factors varies. As we discuss, the consistency and predictability of the law in this area is open to question. Finally, however, we cautiously suggest that some consistent messages do begin to emerge: the courts’ apparent preference for certainty in diagnosis and prognosis provide pointers for how cases might be decided.

## I. INTRODUCTION

The courts are much inclined to speak of the welfare principle as an absolute standard and an unproblematic concept which can act as a panacea for all ills affecting children. This is not altogether surprising since it is they who get to define its content in any given situation … [I]t becomes apparent that the process fails to establish coherent and consistent principles to govern matters which … are important issues of public policy.^1^

Bainham’s remarks were made in 1987 and were directed specifically towards the judges’ approaches to determining the welfare or ‘best interests’ of children. Despite three decades of judicial and legislative developments, his comment appears no less pertinent today, and not only to judges’ decisions about minors.^2^ In this article, we explore judicial decisions about the best interests of incapacitated adults, specifically those in the minimally conscious state (MCS). Patients with this disorder of consciousness have increasingly come before the courts for a decision about their treatment and, in particular, non-treatment. We explored all of the rulings up to July 2016 we could find in English law involving these patients, with a view to identifying the courts’ approaches to these cases. This is not an arid critical exercise. There are estimated to be 12,000–48,000 patients in the MCS in the UK;^3^ treatment might well continue for many of these patients but there will be some for whom non-treatment is being contemplated, so the messages emanating from the English courts might have import for a great many patients, as well as their loved ones, healthcare professionals and others who are providing them with care and treatment.^4^

Our focus is on describing and illustrating the themes we detected in seventeen of the MCS (and, as will be explained, related) rulings; first, however, we outline the nature of the MCS and the legal framework that governs the (non-)treatment of such patients, before detailing the methods by which we have found these themes.

## II. DISORDERS OF CONSCIOUSNESS AND THE COURTS

Our current understanding of disorders of consciousness can be traced back to the intensive efforts of medical scientists in the 1950s.^5^ Various types of disorder are nowadays recognised, of which three need explanation here. In coma, the patient is unresponsive, his or her eyes are closed and movement is limited to reflexive responses.^6^ Having been recognised by the Ancient Greeks, systematic diagnosis of coma developed from the mid-19th century.^7^ The vegetative state (VS) was first recognised in 1940,^8^ with the term ‘persistent vegetative state’ (PVS) following in 1972, to refer to those who remained in this state for long periods.^9^ In PVS, patients spontaneously open their eyes in the course of normal sleep and waking cycles, but show no signs of awareness. They show signs of arousal, which may manifest in laughing, crying or violent movement (for example), but this is not in response to external stimuli, so is not consistently reproducible. Nowadays, guidance from the Royal College of Physicians stipulates that VS is to be described as ‘continuing’ (rather than ‘persistent’) if it lasts more than 4 weeks, and ‘permanent’ if it lasts more than 6 months following head injury or 12 months when arising from another cause.^10^

By the 1990s, queries about the prognostic value of a PVS diagnosis and gathering evidence of awareness in some patients diagnosed with PVS was prompting disquiet amongst some of those working in neurological rehabilitation.^11^ The ‘minimally conscious state’ (MCS) emerged as the definitive term to describe some of these patients in a 2002 diagnostic guideline.^12^ Patients in MCS show limited, but consistent and reproducible, signs of awareness. These signs may include visually tracking objects, bodily movements, vocalisations and speech, and diagnosis is generally made using the Sensory Modality Assessment and Rehabilitation Technique (SMART) and the Wessex Head Injury Matrix (WHIM) tools.

Given their limited consciousness and awareness, patients in the VS or MCS require support from professional, as well as unpaid or familial, carers. As they are unable safely to eat or drink by conventional means, patients will be reliant on clinically-assisted nutrition and hydration (CANH) ie some form of tube feeding. Feeding tubes will sometimes become displaced or need to be replaced, necessitating decisions about whether or not re-insertion should occur. And sometimes those close to the patient will seek to have tube-feeding withdrawn.

The law obviously has a stake in such life-or-death decisions. Indeed, medical law has a longstanding interest in disorders of consciousness and the dilemmas associated with treating patients with such disorders. The New Jersey Supreme Court ruling in *Quinlan* in 1976, which concerned the (non-)treatment of a patient in the PVS, is arguably one of the key starting points in the development of this distinct field of law.^13^ In the past two decades, decisions like these have increasingly come before the English courts, with numerous high-profile rulings addressing the treatment that such patients should (not) receive.

English law first took a position on the withdrawal or withholding of life-support from patients in the PVS in the *Bland* ruling in 1993.^14^ Breaking legal ground in this jurisdiction, the Lords decided that the withdrawal of CANH was permissible in the ‘best interests’ of Anthony Bland. The Lords signalled that future such cases would require judicial determination, a requirement that remains in place,^15^ despite the misgivings of some commentators.^16^ Indeed, *Bland* remains the leading ruling in this area, notwithstanding the changes effected by the Mental Capacity Act 2005,^17^ which came into force in 2007. Of course, *Bland* concerned a patient in the (P)VS. MCS was first mentioned in court in 2002, in *A v H*,^18^ in which a neurologist posited a diagnosis of MCS in a PVS patient, due to the presence of features incompatible with the diagnostic guidelines for PVS. A declaration approving the removal of CANH was nevertheless issued. This is not the only case to have come before the courts in which some of the criteria for diagnosing PVS have not been met, but the diagnosis was nevertheless accepted, and treatment withdrawn.^19^ However, MCS has since been confronted squarely,^20^ and further cases can be anticipated because, since 2007, the withdrawal of CANH from these patients must also be decided in court.^21^

The courts’ decisions in this area are nowadays governed by the Mental Capacity Act 2005. According to this Act, incapacity involves the inability to make or communicate a particular decision due to ‘an impairment of, or a disturbance in the functioning of, the mind or brain’.^22^ Capacity is to be presumed,^23^ but this may be found lacking where the individual is unable ‘(a) to understand the information relevant to the decision, (b) to retain that information, (c) to use or weigh that information as part of the process of making the decision, or (d) to communicate his decision’.^24^ A patient who lacks capacity can seek to determine (or at least influence) his or her future treatment by making an advance decision to refuse treatment or by conferring a lasting power of attorney.^25^ Recipients of the latter are, however, required to make their decisions in the patient’s best interests—and this is the standard that generally governs if the patient has not conferred such a power or issued an advance decision. The Mental Capacity Act sets out a checklist of factors to consider when determining the best interests of incapacitated adults, which requires reference to ‘the person’s past and present wishes and feelings (and, in particular, any relevant written statement made by him when he had capacity)’, ‘the beliefs and values that would be likely to influence his decision if he had capacity’, and ‘the other factors that he would be likely to consider if he were able to do so’.^26^

We sought to explore how the courts approach such best interests decisions, specifically when dealing with MCS patients. Before outlining the themes we detected, we will first explain the methods by which we located the relevant rulings and undertook our analysis thereof.

## III. EXPLORING THE JUDGMENTS: METHODS

This article forms part of a larger project, which seeks to explore the way(s) in which ‘best interests’ manifest, coincide and diverge in medical law and bioethics.^27^ The project focuses on ‘best interests’ in the courtroom and, in particular, in judges’ rulings. Legal judgments capture the judge’s summary of the case and decision thereon. As such, they are written by the judge for the consumption of the affected parties, as well as interested observers and the wider public. Of course, these are not verbatim transcripts of the entire court proceedings. While such a transcript contains all of the evidence, including witness testimony and cross-examination, a judgment is necessarily more selective: the judge decides which evidence to include, which precedents to draw on, and which areas of the narrative to emphasise. Fuller transcripts can be obtained at a cost, but judgments tend to be publically available and are an appropriate focal point, as these express and interpret the law.

Our aim was to focus on a discrete cluster of judgments, as this was practicable and would enable us to develop and test methods of research that occupy the interface of law, bioethics, and qualitative research. Reasoning that the methods involved in ethico-legal analysis are sometimes opaque, we sought systematic methods that would add transparency and reproducibility to our analysis.^28^ We chose as our cluster of rulings those determining the treatment or non-treatment of people in MCS, including those patients for whom this was a possible diagnosis. In the rulings we identified, the withdrawal or withholding of life-supporting treatment was in issue, and in particular CANH.^29^

We chose to focus on MCS cases because the rulings are topical, appear relatively novel, and were few enough in number to allow thorough analysis within the time available. In order to obtain the rulings, Westlaw and Lexis-Nexis databases were searched in December 2015 (and again in July 2016) using the keywords ‘minimally conscious state’, ‘vegetative state’, ‘treatment withdrawal’, and ‘artificial nutrition’.^30^ At the same time(s), the *Medical Law Review* online database was searched for relevant related literature, and the resultant list augmented by existing literature reviews conducted for the authors’ prior research in this area. Snowball searches captured additional rulings that were cited in the references or commentaries.

In total, 17 rulings were found, including seven in which MCS was either queried or a diagnostic possibility on the facts of the case (Table 1).^31^ The judgments were imported into NVivo 10, a software package that aids the analysis of textual sources. Thematic analysis was used, a method commonly deployed for the analysis of social science data in order to discover ideas and concepts that occur across a set of sources.^32^ While typically associated with qualitative analysis of empirical data (from, for example, interviews or focus groups), this sort of method has been used to analyse documentary sources, for example in the context of qualitative meta-analysis,^33^ as well as, pertinently, analyses of Hansard, media reports and Supreme Court rulings.^34^ To find themes, we adapted the method set out by Braun and Clarke:^35^ The judgments were closely read and ‘codes’ relating to frequently occurring words, phrases, and topics devised inductively and applied line-by-line. The themes reported here were discovered by comparing and aggregating these codes with other codes containing similar concepts, a process that was repeated iteratively until broad themes were developed that could be traced back to the original text. Broad descriptions of themes were written, with direct quotations selected to illustrate each theme,^36^ and the entire judgments were then re-read to ensure the themes were faithful to the original context. Analysis was primarily undertaken by the second author; however, to ensure consistent coding, four of the judgments were coded in parallel by both authors, and consensus reached about the applicability of particular codes. Themes were regularly discussed as the analysis developed.

**Table 1.** Cases germane to Minimally Conscious State

Ruling	Court	Diagnosis	Order sought	Outcome
*Frenchay v S* [1994] 2 All ER 403	CA	Possible MCS	Withhold CANH	Granted
*Re D* [1997] 38 BMLR 1	Fam	Possible MCS	Withdraw CANH	Granted
*Re H* [1998] 3 FCR 174	Fam	Possible MCS	Withhold CANH	Granted
*NHS Trust A v H* [2002] 1 FCR 713	Fam	Possible MCS	Withhold CANH	Granted
*W v KH* [2004] EWCA 1324	CA	Possible MCS	Continue CANH	Granted
*W v M* [2011] EWHC 2443	COP	MCS	Withdraw CANH	Rejected
*Re JD* [2012] EWHC 4420	Fam	Queried MCS	Withdraw CANH	Granted
*NHS Trust v L* [2013] EWHC 4313	Fam	MCS	Withhold CPR	Granted
*Aintree v James* [2013] UKSC 67	SC	MCS	Withhold LST	Granted
*NHS v VT* [2014] COPLR 44	COP	MCS	Withhold CPR	Granted
*Sheffield v TH* [2014] EWCOP 4	COP	MCS	Continue LST	Deferred
*County Durham v PP* [2014] EWCOP 9	COP	MCS	Withdraw CANH	Granted
*Lincolnshire v N* [2014] EWCOP 16	COP	MCS	Withhold CANH	Granted
*Gloucestershire v AB* [2014] EWCOP 49	COP	Queried MCS	Withdraw CANH	Granted
*St George’s v P* [2015] EWCOP 42	COP	MCS	Withhold LST	Rejected
*M v N* [2015] EWCOP 76	COP	MCS	Withdraw CANH	Granted
*Re S* [2016] EWCOP 32	COP	Queried MCS	Withdraw CANH	Granted

**Key**: CANH = *Clinically-assisted Nutrition and Hydration*; CA = *Court of Appeal*; COP = *Court of Protection*; CPR = *Cardiopulmonary Resuscitation*; Fam = *Family Court*; LST = *Life Sustaining Treatment*; MCS = *Minimally Conscious State*; SC = *Supreme Court*

Two notes on the nature of our work are warranted. First, the analysis here is qualitative, as opposed to quantitative. We have nevertheless provided some quantitative information, such as the number of times a code was identified and the number of rulings in which this occurred. Such an approach should add transparency (thereby hopefully making a modest contribution to doctrinal scholarship). However, the quantitative information should be treated cautiously. Coded passages may vary in length and coding may be revisited as themes emerge. Ultimately, the focus should be on the themes, rather than the codes. Secondly, qualitative data are produced using a naturalistic, rather than positivistic, paradigm. In other words, rather than collecting objectively reproducible statistical information, qualitative work collects the subjective impressions of the researcher(s) about the meaning, inference and tone of the content under examination. Sampling strategy is also important, as samples are usually purposively selected for relevance and not representative of a wider whole, so not typically generalizable. Our rulings are but a sample of those determining the best interests of incapacitated patients, but we notably sought to capture the entire published population of cases that specifically concerned those in the MCS. Inevitably there will be (perhaps many) unreported cases. As such, it is an interesting question whether our findings might be generalizable, even if only to future cases of this specific sort. Drawing on our findings, we later comment on the predictability of future rulings, which suggest some efforts at generalisation. We leave it to the reader to judge whether this is a defensible strategy.

Two of the emergent themes (‘Facts, Evidence and Experts’ and ‘Judicial Approaches and Processes’), which were based on aggregating 21 codes, are reported here (Table 2).^37^ Our question in this article is: what approaches do the courts take to these cases? The answers arising from our themes have been organised into the following five sections: a decision for a court; heeding the experts; rewarding consensus; striking a balance; and seeking certainty.

**TABLE 2.** Codes and Themes

Code no.	Code name	Summary of code description	No. codes	No. cases/17	Theme	Article section
1.	Tragedy	Emphasis on tragic nature of situation	20	8	Judicial approaches and processes	IV
2.	Disputes	Full-blown disputes, not mere differences of opinion (eg between experts)	26	7	Judicial approaches and processes	IV
3.	Temporal issues	/lack of urgency of decision, discussions of timeliness	18	7	Judicial approaches and processes	IV
4.	Uniqueness	Each case judged individually, as circumstances and personal effects differ between cases	6	4	Judicial approaches and processes	IV
5.	Chronology	Simple time-points that feature in the case	104	16	Facts, evidence and experts	IV
6.	Family expertise	Evidence from families, friends, partners or other close associates and its status	39	14	Facts, evidence and experts	V
7.	Medical expertise	Evidence from medical witnesses and its status	104	17	Facts, evidence and experts	V
8.	Medical self-determination	Compelling (or not) doctors to treat	22	4	Facts, evidence and experts	V
9.	Non-doctor expertise	E.g. nurses, occupational therapists	40	11	Facts, evidence and experts	V
10.	Objective expertise	Expert opinion that is leant objectivity by the use of objective measurement, and/or appeals to inter-subjectivity	41	10	Facts, evidence and experts	V
11.	Shared decision	Instances of shared decision-making	5	3	Judicial pproaches and Processes	VI
12.	Reasonableness	Using reasonableness as a criterion for judgment (eg reasonable patient standard)	4	3	Judicial approaches and processes	VI
13.	Consensus	Presence/absence of broad agreement about a particular state of affairs or idea	22	12	Judicial approaches and processes	VI
14.	Compromise	Judicial attempts to forge compromise	1	1	Judicial approaches and processes	VI
15.	Balancing exercise	Discussion of how elements are balanced, and of balance sheet approach per se	45	11	Judicial approaches and processes	VII
16.	Level of consciousness	(Lack of) evidence of higher brain function; relationship of brain function to being a person; suggestion that lack of sentience vitiates personhood; presence or absence of PVS or MCS	184	17	Facts, evidence and experts	VIII
17.	Misdiagnosis	Not getting the diagnosis right, medical errors	25	10	Facts, evidence and experts	VIII
18.	Changing	Features of the case which are (or have been) subject to change (eg medical advances, the law, the opinions of participants)	14	3	Facts, evidence and experts	VIII
19.	Clinical prognosis	Recovery prospects, markers of improvement, measures of decline and stasis; plans for future treatments	184	17	Facts, evidence and experts	VIII
20.	Uncertainty	Of events, personality, illness etc.; difficulties in making predictions; unpredictability; imprecision; lack of definition; blurry concepts	83	13	Facts, evidence and experts	VIII
21.	Natural and unnatural	Artificiality or un/naturalness of life-sustaining treatment and death	21	9	Facts, evidence and experts	VIII

## IV. A DECISION FOR A COURT

The first notable approach taken by the courts in MCS cases involves the judges framing the cases before them in such a way that a judicial decision is required. There are five features of these rulings that create this framing.

First, the judges often refer to the *tragic* nature of the case—‘This is another of the very sad cases of a patient who has suffered very serious brain damage’^38^—and will express their sympathy for the family.^39^ Tragic cases, we might presume, are likely to be difficult cases. Of course, not every difficult case involving the care of an incapacitated patient will reach a court; however, the recurrence of these remarks begins to convey the sense that the judges feel there is a role for them to play here.

That sense is strengthened by those cases in which, secondly, the judges highlight any *disputes* arising in the case, such as disagreements between the clinicians and the family. Conflicts are, of course, routinely the court’s business, and some level of dispute might be expected given the adversarial nature of law. The judges will nevertheless occasionally censure overly adversarial parties. For example, Cobb J appears critical of the patient’s son-in-law in *PP*, who ‘devoted significant time and energy into collating evidence for a potential civil action, which I cannot but observe has detracted a little from the key issues engaged in these proceedings’.^40^ Clinicians have also been criticised for impeding the settlement of a dispute, by failing to give ground to a family that has ‘politely and cogently articulated’ its opposition.^41^ By conveying the existence and persistence of such disputes, the courts appear to reinforce their standing as the appropriate forum in which to resolve these cases.

This standing can be further reinforced in, thirdly, the *detailed chronology* that is provided by the judge, which traces the course of the patient’s condition, the investigations undertaken, visits by expert witnesses, and the like. In *H*, the relevant interval was 8 years and everyone—professionals, family members, and judge—agreed that withdrawal of treatment was appropriate.^42^ Here, the history of the patient, dates of examinations and relevant reports are conveyed in just six paragraphs.^43^ Contrast this with *W v M*: the relevant interval was also 8 years but here opinions about withdrawal were split, and twenty-one paragraphs are devoted to the clinical narrative.^44^ The length of these accounts seems indicative of the difficulty involved in the case: the longer the account, the greater the apparent unease of the protagonists, and arguably the greater the need for judicial appraisal.

Fourthly, the *urgency* of the decision will also be highlighted where appropriate. On occasion, the judges are critical of the time that has been taken to bring a case to court, if this means that the patient’s ‘rights are compromised in consequence of avoidable delay’.^45^ Where a case is deemed urgent, then this may incline the courts to expedite matters. *Frenchay* is particularly notable in this regard: the parties were in the early stages of seeking withdrawal of CANH, but the dislodgement of the patient’s feeding tube created a sense of urgency; the court authorised the withholding of further feeding, despite the patient not fully meeting the VS diagnostic criteria.^46^ This construction of a sense of urgency has occurred since,^47^ and perhaps questionably so. The orthodox position is that withholding and withdrawing are to be treated as legally synonymous. In cases like *Frenchay*, the courts appear to seize the opportunity to withhold, rather than withdraw, as a matter of urgency. If, however, the two are synonymous, then the urgency arguably disappears—the feeding tube can be reinserted and then later withdrawn, if the court so decides.^48^ A sense of urgency nevertheless helps to shore up the court’s position as the rightful decision-maker.

Finally, in four of the cases, the judges referred to the *uniqueness* of the case before them.^49^ For example, in *VT*, Hayden J notes that ‘every case is different’,^50^ and he cites Hedley J in *Wyatt*: ‘The infinite variety of the human condition never ceases to surprise and it is that fact that defeats any attempt to be more precise in a definition of best interests’.^51^

Taken together, these observations seem to make the case that the withdrawal or withholding of life-supporting treatment from patients in the MCS is properly a decision for the courts. Perhaps these framings reveal that the judges are aware of the criticism, levelled particularly at CANH decisions for PVS patients,^52^ that judicial oversight is required, and they are seeking to undergird their power to decide. In short, a tragic, contested, urgent, complex, and unique case is a matter for the judges. Of course, the mere presence of such factors in a case reveals nothing about the decision that might then by taken by the judge. We begin to get closer to this by looking to the ways in which the courts view the different types and forms of expertise that enter the courtroom in these cases.

## V. HEEDING THE EXPERTS

Outwardly at least, the law seeks to empower the patient.^53^ Of course, as they lack capacity, patients in a minimally conscious state cannot presently articulate their wishes and preferences, at least in any direct and unmediated sense. When a patient lacks capacity, an advance decision to refuse treatment offers the most direct and powerful expression of such wishes that a court might encounter; less directly, but also importantly, the court might hear such wishes via the opinion of the donee of a lasting power of attorney. However, neither of these legal instruments were enacted by the patients who featured in our judgments. As such, in these cases, other people (or types of evidence) claim to speak for or on behalf of the patient, but in all cases the patient’s views are mediated by these ‘others’. These ‘others’ implicitly appear to claim some sort of ‘expertise’ about or over the patient. Here, we draw out four distinct types of expertise on which the judges tend to reflect when reaching a judgment. It is apparent that the different types of expertise—and types of ‘expert’—exert varying degrees of influence over the final decision that is reached.

*Family expertise* featured in fourteen of the seventeen rulings, with the judges reflecting on the experiences and views of the patient’s family, as a step towards reaching their decisions. Of course, under both the Mental Capacity Act and the preceding law, the court’s concern must be with the best interests of the patient, arguably irrespective of the family’s views *per se*. Certainly, such views should nowadays be sought as a means of finding the patient’s best interests—and they must be sought and heeded if the family member in question has a lasting power of attorney (which, as noted, was not the situation in any of the cases here). However, these views do not have freestanding weight: relatives can indicate what they think the patient would say if he or she could be consulted,^54^ or otherwise indicate the values of the patient,^55^ but the key consideration is indeed the best interests of the patient, not the views and values of the family.

This is borne out in the present cases, since a family’s views appear at most to lend supplemental weight to a decision being taken in a particular direction. As such, in *A v H*, Butler-Sloss P states:

The family, for whom this has been over many years an extremely distressing experience, have said for a number of years that they did not wish their mother to continue to be artificially kept alive and that they would wish this to come to an end. Therefore the family is entirely in agreement with the application of the hospital.^56^

This comment is striking, since the statement is not linked to the (past) wishes of the patient, which is how family views would nowadays be framed. But note that the family’s views seem only to gain weight insofar as they accord with the views of the doctors.

The (merely?) supplementary value of family views is also evident elsewhere, in cases in which their views are attached to something other than the doctors’ views. In *P*, Newton J focuses on a dispute between the family and the hospital:

In looking at those aspects and as to whether or not P would assess his life as being regarded as worthwhile I attach far more weight to the relevant expressions of his articulate and well informed family members and friends who have direct knowledge of P’s pre-injury knowledge, understanding and philosophy, in particular those who know about his beliefs and values.^57^

Here, while the family perspective is firmly favoured over that of the medical, the statement allies the family view with that of the patient’s past values. Within these cases then, the family view is not sufficiently influential on its own account.

Notwithstanding Newton J’s misgivings in *P*, it is overwhelmingly apparent in all of the cases that *medical expertise* carries a great deal of weight with the courts. The special qualification and experience of the doctor is frequently given special prominence, as illustrated by *Frenchay*:

The consultant in question, whose curriculum vitae is before us, is a consultant of very wide and long experience in the treatment of the acutely disabled, including the young acutely disabled. It is apparent from his curriculum vitae that he has the most extensive and wide ranging experience in this country and abroad. He is extremely well qualified in medical terms and he has also, perhaps relevantly, engaged himself in the consideration of ethical questions.^58^

The latter idea that doctors might have some form of ethical expertise is occasionally detectable in subsequent rulings, on occasion being mentioned explicitly,^59^ elsewhere featuring implicitly.^60^ What features more regularly in the judgments is an emphasis upon those occasions when the medical professionals are in consensus. For example, in *M v N,* Hayden J notes:

There is complete agreement between the doctors that Mrs. N is suffering from very advanced Multiple Sclerosis. Given that this is a degenerative disorder the concept of rehabilitation has strikingly limited utility. Whilst some pragmatic adjustments could be made to improve the very limited quality of Mrs. N’s life, when these were analysed properly, all agreed they could accurately be characterised as palliative care.^61^

While consensus between doctors might appear to influence the judges, it is notable that the degree of consensus between family members appears to attract no comment.

Evidently, a different degree of weight is accorded to medical views as opposed to family views. Indeed, sometimes the family view may be in need of correction by the clinicians. For example, in a speech about the relative weight of expertise in *M v N*, Hayden J warns:

family members may sometimes interpret simple reflexive movements as more positive interactions. They need information and support from clinicians who can explain what behaviours to look for.^62^

Doctors may correct families, but the judges seem less inclined to suggest that families might correct doctors.^63^ It is certainly made apparent in some cases,^64^ following previous case law,^65^ that a doctor cannot be compelled to give treatment against his or her clinical judgment:

That, in my judgment, is not only medically contrary to his best interest, it is difficult to reconcile with the underlying theological premise that the family advances. It can hardly be right to expect doctors to cause pain for no justifiable medical reason other than to accommodate the religious or other beliefs of a patient.^66^

Such a principle suggests not only that doctors have an epistemically favoured position, but also that their professional status grants them particular privileges as well.

*Non-medical expertise*—such as is offered by nurses, allied health professionals like physiotherapists and occupational therapists, or full-time care assistants—receives a rather more mixed treatment. The most favourable account of such evidence was provided by Baker J in *W v M*. Here, the testimony of the patient’s professional carers was ultimately favoured over that of the patient’s family:

Like Professor Turner-Stokes, I wondered when I first read the papers whether the carers were over-interpreting M’s behaviour, seeing what they wanted to see. Professor Turner-Stokes has come to accept that the carers’ accounts are broadly accurate. Unlike Professor Turner-Stokes, I have also the benefit, not only of reading the carers’ statements, but also listening to them give oral evidence over a number of days. I have been impressed, indeed moved, by their professionalism and dedication to their demanding job. Although most of them hold a clear view that ANH should not be withdrawn in this case, I find that they have remained objective in the evidence they have given and that their accounts are reliable and accurate.^67^

Along with nursing staff, carers like these are likely to have the most contact with the patient. However, the judges rarely afford the evidence of allied health professionals such prominence or assign it such positive value as it was granted in *W v M*. More often, allied health professional testimony is confined to brief acknowledgements that these witnesses are in agreement with the doctor or, more rarely, have expressed dissent:

she may gain pleasure from things which one of her care home carers (albeit that her close family members believe differently) consider she has derived pleasure - company, some television programmes, some physical touch.^68^

Such expressions of dissent are often considered in a cursory manner:

The entries in certain of the nursing records to which I have been referred have not in fact been substantiated in any meaningful way by the repeated examinations of the numerous medical experts.^69^

When, in the rare case, allied health professional testimony does acquire prominence, it is usually because this needs to be corrected. In *Re JD*, for example, the physiotherapy team that challenged a VS diagnosis was judged to have ‘innocently misinterpreted’ the patient’s vocalisations.^70^

The final source of expertise to which the judges frequently refer is what we term *‘objective’ expertise*. Here, we refer to the evidence provided by empirical assessment ‘tools’, such as SMART and WHIM, which systematically measure the responsiveness of the patient. The courts have endorsed the use of such tools since the decision in *W v M*, where Baker J suggested that the outcome in that case, that treatment continue,

demonstrated the crucial role played by the formal assessment tools, the SMART and the WHIM. The history of this case shows how cases may be misdiagnosed if these tools are not used.^71^

While the use of such tools may be ‘crucial’, the judges have nevertheless resisted the idea that the tests’ findings will be determinative. Even the very use of these tools must be balanced against other factors, in view of the delays their use might entail:

This is not to say that assessments ought to be rushed or that delays may not sometimes be clinically purposive, but respect for a patient’s autonomy, dignity and integrity requires all involved in these difficult cases to keep in focus that these important rights are compromised in consequence of avoidable delay.^72^

Moreover, as Hayden J points out in *M v N*, the findings should not be treated as determinative because the tools are not entirely ‘objective’. He compares the veracity of the tool’s findings to the reports of family members, noting that the assessments are

susceptible to inbuilt professional bias. Professional enthusiasm and determination are admirable qualities and are to be nurtured, but it is important to guard against overly optimistic assessment driven by a vocational desire to try to make a difference. These assessments tools have an inevitably subjective complexion to them.^73^

Assessment tools may be a ‘crucial’ aid, but it appears that that is all they are – an aid to assessment and, by extension, an aid to judgment as to what should or should not happen in terms of the patient’s care.

In sum, the judges will appraise the different types and forms of expertise that enter the courtroom and, in doing so, they display a clear preference for (seemingly) ‘objective’ tests and, in particular, the views of the doctors. However, this is not a consistent finding—sometimes other evidence, arising from other forms of expertise, appears to assume prominence. The message appears to be that judgment is a matter for the court. That, of course, is implicit in the very nature of judging, so may be unsurprising. But it does not yet tell us enough about the approaches the judges will take or the decision that might ultimately be made. To get closer to these, we must move to our next section, which discusses the judges’ indications about the approaches on which they look favourably and unfavourably.

## VI. REWARDING CONSENSUS

The judges’ approaches to these cases is further revealed in the comments that they make about the approaches that are taken by others, specifically the relevant protagonists involved, or with an interest, in the patient’s care. These comments emphasise co-operation, consensus, compromise, and the reasonableness of the parties.

Starting with *co-operation*, the judges are occasionally inclined to urge the parties to work together:

So far as the future is concerned, there must be a radical review of M’s care plan. I firmly believe that this is a process that should, if possible, be carried out by family members and professionals working together. The Court of Protection is here to help with those endeavours if necessary, but in the first instance, I urge all parties to try to agree a plan for M's future care.^74^

The judges have praised a co-operative approach, in which (for example) the doctors communicate openly with family members and adjust their plans in light of family input.^75^ This apparent preference for collaboration reflects wider ethico-legal support for ‘shared decision-making’.^76^

Implicit in this apparent preference is the idea that co-operation is a reasonable way to proceed. Sometimes the judges will explicitly remark on the *reasonableness* of the parties or the evidence. Reference was made to the reasonableness of the patient’s chances of survival in *L*.^77^ The latter sort of reflection is invited by the Mental Capacity Act, as the Code states that ‘All reasonable steps which are in the person’s best interests should be taken to prolong their life’.^78^ The Act itself requires the assessment of the patient’s best interests to involve, ‘so far as is reasonably ascertainable’, reference to the patient’s views and values.^79^ However, in *Aintree*, the Supreme Court, disagreeing with the Court of Appeal, emphasised that it is *the* patient’s views and values that should be considered, not the views and values of the *reasonable* patient.^80^ Presumably, the patient’s views and values can be reasonable or unreasonable, but, in their dealings with other protagonists and evidence, the courts otherwise appear to favour reasonableness. We have already seen some of the judges’ aversion to adversarial parties. Elsewhere, we see the courts effectively commending the reasonableness of those involved, as occurred, for example, in *Frenchay*, where the reasonableness of a witness’ opinion was noted: ‘the conclusion at which S’s consultant had arrived was reasonable and bona fide’.^81^

The judges’ preference for reasonable co-operation between different parties might imply that the courts will endorse any *consensus* that might then result. Certainly, evidence that is unanimously agreed, particularly amongst expert witnesses, can prove conclusive in court.^82^ Moreover, the position of the Official Solicitor might acquire special significance, if it accords with one of the parties,^83^ and perhaps particularly if the Official Solicitor changes his or her mind having heard all of the evidence.^84^ However, the unanimity of experts is not always sufficient to determine a case. For example, the Supreme Court in *Aintree* endorsed the trial judge’s rejection of the medical opinion,^85^ despite this having the backing of the Official Solicitor.^86^

Sometimes, rather than endorse a position offered by some or even all of the parties, the judges seek to reach their own decision, which on one occasion sought to capture a *compromise*. This occurred in *VT*, a case in which the family wanted to continue treatment of the patient in all circumstances, but the doctors favoured non-treatment.^87^ Hayden J offers a compromise by analysing the cardiac and pulmonary components of cardiopulmonary resuscitation (CPR) separately,^88^ and concluding that, while cardiac resuscitation is not in the patient’s interests, (brief) respiratory resuscitation in certain circumstances is permissible. Given that the body’s cardiac and pulmonary systems are so closely intertwined, it is questionable whether such a separation is logically defensible. However, we have argued that the aim of a compromise is to make all the parties feel that they have not only sacrificed a portion of their aims, but also gained something too.^89^ Having made such gains, the parties might then be more satisfied with the solution. Hayden J’s solution looks like such a compromise,^90^ especially when his rationale is explained: ‘to prohibit CPR only in the event of cardio collapse, in my view [would] be too blunt; moreover, it seemed to me to be likely to cause further dispute and perhaps a return to this court’.^91^

As such, the judges might evince a predilection for reasonableness and for co-operation, but it is not necessarily going to be the case that any consensus reached by the parties will be endorsed by the court. No matter how united the parties might appear, we again gain the sense that the judges retain the final right of say. Sometimes they will authorise the course favoured by the parties; elsewhere, they will adopt a different course, which might capture a compromise between the different parties. While the judges seem to have a preference for *the parties* approaching these cases in particular ways, we do not yet have a clear idea of *the judges’* own approaches to these cases. These approaches become clearer in our next, major, section, in which we focus squarely on the judge’s key concern, which is with the best interests of the patient.

## VII. STRIKING A BALANCE

In order to reach their decisions about these incapacitated patients, the judges must determine where the patient’s best interests lie. When approaching this task, the judges have long been inclined to undertake a balancing exercise, which involves drawing up factors for or against the proposed course, before reaching a conclusion. As Thorpe LJ put it in a ruling from 2000, ‘the task in each case is to balance all the relevant factors and to decide what are the best interests of the person unable to make his own decision’.^92^ Thorpe LJ may have suggested that this would occur in ‘each case’, but (speaking extra-judicially) Baker J has recently indicated that this is not a uniform requirement as such; rather, ‘the court will *often* adopt a balance-sheet approach’.^93^ Baker J’s qualification is amply borne out in our cases, where we see the judges taking different approaches to the assessment of the patient’s best interests: while some judges provide an explicit ‘balance sheet’, others do not.

In eleven of the seventeen cases found within our search period, no balance sheet is provided in the judgment. In eight of these cases, the omission is explicable. In seven rulings, the court decided that the patient was in a VS; no balancing exercise is undertaken in such cases.^94^ In the eighth case, in which an MCS diagnosis was accepted, the judge felt that further assessment of the patient was needed, so no ruling was made on the best interests of the patient.^95^ This leaves three cases—in all of which MCS was the accepted diagnosis—in which a balancing exercise was not undertaken, or at least not articulated. In the first of these, the judge provides no reason for not undertaking an explicit balancing exercise.^96^ More revealing, in terms of the courts’ approaches, are the remaining two cases. In these rulings, the (same) judge declined to strike a balance, as he expressed discomfort with the very idea of seeking to balance different factors. In *VT*, Hayden J felt that, while considerations of ‘the intrinsic value of life itself’ and of ‘pain and indignity … can be juxtaposed, they cannot, to my mind, really be balanced. We are not comparing like with like’.^97^ He made this sort of point more forcefully in *M v N*: ‘The exercise is almost a balance of opposites: the philosophical as against the personal. For this reason, … I consider that a formulaic “balance sheet” approach to Mrs. N’s best interests is artificial’.^98^

This leaves six cases in which balance sheets were explicitly provided.^99^ In order to try to perceive the judges’ approaches, it is worth spelling out the factors cited in these rulings, as well as the decisions reached. Starting chronologically with the 2004 case of *KH*, the balance sheet is one-sided and succinct: on the side of continuing CANH are the suggestions that survival may be beneficial and starvation less dignified than if KH were to succumb to her condition.^100^ In that case, continued treatment was authorised.

Four or more factors on each side are provided in the five later cases. In *W v M* in 2011, seven factors were cited in favour of continued treatment: respect for the sanctity of human life; the pain and distress that might accompany starvation and dehydration; M will continue to experience sentient life; M may continue to take pleasure from life; M’s pleasure can be enhanced with improved stimulation; M’s pleasure could be increased by improved surroundings; and M can be expected to live for some years.^101^ Weighed against these were nine factors pointing towards the removal of CANH: M’s current and anticipated pain and discomfort; the unpleasant effects of treatment; evidence of M’s distress; the indignity of M’s situation; M’s wishes and feelings prior to her collapse; the family’s assessment of what M would want; the family’s assessment of M’s best interests; the unlikelihood of M’s recovery from MCS; and the fact that efforts can be taken to make M’s death as comfortable as possible.^102^ Baker J decided the former factors outweighed the latter, and ruled in favour of continued CANH.

In contrast to Baker J, in the next three cases the judges preferred a decision not to treat. In *L* in 2013, the issue was whether or not CPR should be withheld. In favour of providing CPR were seven factors: L was comparatively young and medically stable; the sanctity of human life; L was aware and deriving comfort from his family; L’s health had improved in the preceding four months; this would be in line with L’s wishes, feelings, beliefs, and values; this was what L’s family judged to be in his best interests; and L’s dignity would be honoured and his autonomy promoted.^103^ However, Moylan J sided with the 11 factors against undertaking CPR: L’s brain would be unlikely to recover further or significantly; CPR would be unlikely to succeed and would cause L harm; the decline that would precede CPR would be likely to cause further brain damage and lessen L’s prospects of recovery; this decline would be likely to cause further physical damage; although L might judge it acceptable, his quality of life would be likely to deteriorate following cardiac arrest; no doctor would be likely to be willing to offer CPR; L was experiencing pain; improvements to L’s health were only modest; L had endured two or three cardiac arrests in 8 months; L’s recovery was unlikely and ill-health expected given his significant co-morbidities; L was vulnerable to infection; and continued intensive care would be invasive, painful, and distressing.^104^

CPR was also one of the treatments at issue in *Aintree* in 2013, where a briefer balance sheet was drawn up at first instance.^105^ In favour of treatment were: the sanctity of life, and the possibility that treatment might prolong David James’ life; James’ quality of life, from which he gains pleasure, and evidence of some improvements in his condition; the likelihood that James would want treatment up to the point it became hopeless; the family’s belief that the point of hopelessness had not been reached; and that it would be wrong for James to die ‘against a background of bitterness and grievance’.^106^ Against treatment were said to be the following factors: James’ body and brain had sustained severe damage, he was unlikely to recover independence, and ‘the current treatment is invasive and every setback places him at a further disadvantage’; the treatment may not work; the treatment would be burdensome; and it was not in James’ ‘interests to face a prolonged, excruciating and undignified death’.^107^ The trial judge favoured treatment and was ruled not to have erred in his approach by the Supreme Court. Nevertheless, the Supreme Court ruled that withholding treatment had been appropriate on these factors, since James had deteriorated further by the time of his death.

In *Lincolnshire* in 2014, the issue was whether CANH should be withheld. Factors in favour of CANH were: if successful, N would live for many more years, and relatively comfortably so; N would be spared the effects of non-treatment and associated risks; N would continue to experience life as a sensate being with some awareness; likely beneficial treatments could be reinstated; and N might continue to gain some pleasure from life.^108^ Pauffley J, however, ruled that CANH should be withheld, because these factors were outweighed by: the likely future pain and distress to N; the invasiveness of the procedures, which would occur under restraint; the risks associated with inserting the feeding tube and of re-feeding syndrome; the need for repeated restraint or continuing sedation; the indignities of N’s situation; the apparent alignment with N’s wishes and feelings; and because withdrawal of CANH aligned with what N’s family believe she would have wanted.^109^

Finally, there is *St George’s v P* from 2015, in which Newton J ruled in favour of continued haemodialysis. He was most persuaded by the following factors: P’s life would be preserved, which is what he would have wanted; withdrawal of treatment would cause P’s death within days; P showed clear responses to his family and friends; and treatment ‘permits improvement of increased awareness to develop, if it can’.^110^ These outweighed the considerations that: some patients die during this treatment; P’s life expectancy was already reduced; treatment is undignified and may be painful or uncomfortable; P lacked independence; and P likely had no potential for ‘meaningful’ functional recovery.^111^

Two striking points emerge from these findings. First, even in cases in which the patient is confirmed as being in a MCS, the judges do not consistently undertake a balancing exercise. Here, we can query whether some consistency in approach—ie drawing up a balance sheet or not doing so—is desirable. However, secondly, it is also apparent that consistency in approach will not necessarily mean consistency in outcomes. In those six MCS cases where balance sheets were provided, the judges did tend to point to similar factors, both in favour of and against a proposed course. On the side of treatment, we see recurring references to the importance of preserving life, the patient’s (positive) quality of life, the patient’s dignity, and the wishes of the patient and/or their family. Some of these factors also feature in the case for non-treatment: for example, there are repeated references to the wishes of the patient and/or their family, the patient’s dignity, and the poor quality of life the patient is enduring or can be expected to endure.

There is, then, some commonality in approach in terms of factors to which the courts are alert, but this does not necessarily mean that outcomes can be predicted: sometimes the judge will opt for treatment, other times for non-treatment. That the balancing exercise is not a *quantitative* exercise is borne out by *W v M*, in which there were nine factors in favour of removing CANH but seven factors in favour of continued treatment, and the latter was the course preferred by Baker J. Evidently, the balancing exercise is *qualitative* in nature. Speaking extra-judicially, Baker J has observed that ‘It is the weight to be attached to each factor that is important rather than the number of factors on each side of the argument’.^112^ This might explain the different decisions: one particular factor might assume particular importance in a given case, as indeed the sanctity of life did in Baker J’s own decision in *W v M*. But we appear no closer to knowing which sort of factor will operate as a trump card in which sort of case. Maybe it would be wrong to expect to know this: as we have seen, the judges are wont to emphasise that every case is different. And, as we have also seen, judges are there to judge. However, the cases evidently have some shared features. More such features emerge in our final section, in which the decisions that the judges might reach start to become more apparent.

## VIII. SEEKING CERTAINTY

Finally, our findings suggest that judges favour certainty, specifically in terms of diagnosis and prognosis, with particular findings here arguably influencing how the court will rule.

We can first detect the judges’ search for certainty in their references to the *diagnostic guidance* issued by the medical profession. Deficiencies in the extant guidance are noted in the early rulings, in which the patient’s diagnosis is not entirely settled. In *H*, for example, the judge suspects ‘that it may be that it is time to review’ the guidance on determining VS.^113^ However, the phenomenon does recur later, after MCS had become a more established diagnosis: in *W v M*, for example, Baker J notes how both experts regarded the relevant ‘guidance as now out of date’.^114^ Such comments serve to convey a situation that is changing and thus a story—of medical science, the law and the patient—that is evolving. It is conceivable that the judges’ comments have helped to build the case for revisiting the guidance: review and revision occurred after *W v M*, for example. However, it is also plausible that the judges’ comments—informed by the evidence presented to the court—merely express (the aforementioned^115^) reservations that are held by the relevant experts at the relevant time.^116^ MCS may have evolved into its own diagnostic category, but it is a notably broad one, perhaps encompassing degrees and therefore a great variety of patients, as indeed the court has noted.^117^ Against such a backdrop, it may not be surprising that the judicial approaches have yet to settle into a coherent whole. Indeed, the relative paucity of published decisions further suggests that the courts have not yet had time to build up a uniform approach.^118^ In short, there may be too few cases and too little scientific certainty for the judges to have settled on the approaches that should be taken and the decisions that should be reached.

But there are indications that the approaches are settling. These indications are provided in the judges’ references to two areas of perceived scientific certainty. First, the judges appear to seek *certainty in diagnosis*—and once the diagnosis is settled, the approach to be taken by the court also becomes more settled. As Newton J states in *P*:

If P is in VS, and there is no real prospect of recovery, then it would not be in his best interests to continue treat him (because a person in a continuing vegetative state has no interests and therefore no best interests). However, if he is in a minimally conscious state then the issue to be considered must be on the balance of best interests in accordance with section 4 MCA and the appropriate professional guidance.^119^

This comment was made in 2015, when the diagnostic guidance had recently been revised,^120^ and the courts had begun to build up an approach to cases of MCS, having already established their approach to patients in the VS. A particular, and certain, diagnosis appears to light the way for the court. By extension, the courts will be alert to any uncertainty in diagnosis and will seek to have this resolved, in order that a decision can be reached.^121^ As one sees in the cases in which MCS was queried but VS was ultimately confirmed, even lone voices of doubt will be heard, and the evidence interrogated, in order to determine which diagnosis (and thus legal approach) is to be preferred.^122^

In short, the legal approach is first determined by resolving whether the patient is in a VS or MCS. Almost without exception,^123^ the outcome for patients in the former group is predictable: life-supporting treatment will be withdrawn or withheld. However, as we saw in the previous section, this is not (yet?) the case for the patients in the MCS: we can increasingly anticipate the sorts of factors that the judges will take into account, but not necessarily the decision that will then be taken.

We nevertheless come closer to being able to make such predictions once we appreciate that *certainty in prognosis* also appears to exert some influence on the outcome of the judgments. A twin concern with certainty in diagnosis and prognosis is evident in Cobb J’s decision in *PP*, in which an expert witness is reported to have described the patient as:

‘somewhere on the spectrum between the vegetative state and an extremely low position on the minimally conscious/minimally responsive state’ with no real prospect of a change in that condition. He felt that P had entered a ‘terminal’ phase of her life, which he described as ‘pre-terminal hibernation’.^124^

In contrast to *W v M*, where treatment was to continue, the judge decided that CANH was to be withdrawn from PP. In cases like *PP*, which culminate in non-treatment, reference may be made to the treatment being ‘detrimental’ if the patient is declining,^125^ or to it being ‘unnatural’, even ‘cruel’.^126^ However there are two particular distinguishing features of such cases that might appear, in terms of prognosis, to explain the different outcomes.^127^ First, there is the patient’s possibility of recovery. Note that, in *W v M*, a witness judged it ‘“highly improbable” that M would emerge from MCS. She placed this probability at less than five per cent’.^128^ M’s chance of recovery might be slight but it is not absent. Contrast this with the early case of *Frenchay*, in which there was some diagnostic uncertainty but the experts felt, after two years of attempting to stimulate the patient, ‘that there was *no* chance of recovery’.^129^ In *M v N*, a similar consensus existed,^130^ while in *P* the door appears to be left open to the possibility of slight recovery.^131^

Secondly, the patient’s proximity to death appears relevant. The patient in *W v M* appeared to have a life expectancy of 10 years.^132^ Contrast this with PP, who was considered to be nearing death (‘pre-terminal’). Other cases in which the imminence of the patient’s death might have nudged the judge towards authorising the removal of life-support include *TH*: although the case did not result in a declaration, it was noted that the patient ‘could die within the next few days/weeks as a result of complications of his neurological condition’ or, in ‘the best case scenario’, would not develop such complications but nevertheless would only endure ‘for several more months or even a year or so’.^133^

As such, the courts seem to invest in scientific certainty. Certainty in diagnosis will light the court’s way, with VS cases dealt with in one way (and leading to predictable outcomes), MCS cases another. Yet, even with MCS cases, which seem generally less predictable, there are prognostic indications that appear to nudge the court towards one decision, rather than another. If the patient has no chance of recovery or the patient is close to death, it seems more likely a decision will be made not to treat.^134^ Doubtless, further rulings are needed to flesh out the factors that will incline the court to non-treatment as opposed to treatment, but there are already some indications available to future litigants, on which basis they can begin to predict how their case will be resolved.

## IX. DISCUSSION

MCS, and the modest pool of rulings determining the fates of such patients, has already sparked a great deal of academic interest, with different scholars exploring the views of those involved in such proceedings,^135^ plus their ethical and legal dimensions.^136^ Rather than replicate such work here, we seek to offer three reflections on our overlapping findings.

First, these judgments are evidently influenced by the ‘scientific’—for which, read ‘medical’—evidence before the court. Some of the findings suggest that the courts are keen to assert their authority over these cases of life of death. This trend is familiar from other medico-legal contexts, where life or death is not necessarily at stake. The ruling in *Bolam*, which found that doctors can look to responsible medical opinion to defend a negligence action, long dominated medical law, both within and beyond its original parameters.^137^ However, the courts have increasingly sought to re-assert their authority in relation to negligence: judges determine whether the body of opinion in question is to be considered ‘responsible’.^138^ This assertion of judicial power certainly emerges from our themes. But what is also striking in our cases, albeit with some exceptions, is that the *Bolam* attitude also appears to be alive and well: in seeking to reach a decision, the judges will look particularly closely at the medical evidence and the opinions of the doctors. Perhaps the judges’ recourse to these experts is explicable because the MCS diagnosis is relatively recent and not entirely settled, and the judges have also only recently begun to encounter such patients: better, perhaps, to heed the medical experts when navigating such terrain.^139^ Whether families (or, perhaps, other carers) will welcome this apparent inclination is, of course, open to question.^140^

Secondly, the rulings seem to offer support to a legal realist, as opposed to a legal formalist, understanding of judicial decision-making. Formalists contend that legal answers to legal problems can be found within the law: extra-legal sources, such as ethical norms, are not needed. Realists, on the other hand, dispute the determinacy of law, ‘insisting that judges primarily decide appellate cases by responding to the stimulus of the facts of the case’.^141^ Few of our cases reached the appellate courts, but the central point appears to stand. Although the extent to which the judges are drawing on ethical norms in these cases warrants further analysis,^142^ the ‘rules’ (such as the one holding that the test is the ‘best interests’ of the patient) appear not to be entirely determinate and the judges seem keen to fix certain (particularly ‘scientific’) facts in order to reach their decisions.

Thirdly, and connected to the problem of indeterminacy, it is apparent that the rulings are not entirely consistent or predictable. Certainly, the approaches we detected revealed a degree of consistency and predictability, but arguably not of the measure that might be anticipated by legal formalists, and what (little?) consistency there is in approach does not guarantee consistency in outcomes. Rather, the message seems to be that a judge will do precisely that—judge—when called on to determine a patient’s best interests.^143^

Whether this is sufficient is open to question. As Birks has noted:

The law … is under constant surveillance. Vigilant critics quite rightly pick over the substance of every judgment. And it is an unrelenting question whether, through time, but allowing for the changes of perception which come with the passage of time, the courts are or are not, can or cannot be, true to the aspiration impartially to treat like cases alike.^144^

For some, like Fuller, this aspiration is central to the rule of law: for him, law is ‘the enterprise of subjecting human conduct to the governance of rules’,^145^ and for law to succeed in this endeavour, it must be consistent in what it says and does.

The greater such consistency there is in the law’s operations, then the more predictable the outcomes of these operations will be.^146^ Interest in the predictability of the courts’ decisions has prompted some researchers to devise

models that can be used to unveil patterns driving judicial decisions. This can be useful, for both lawyers and judges, as an assisting tool to rapidly identify cases and extract patterns which lead to certain decisions.^147^

These particular researchers utilised information technology to help predict decisions of the European Court of Human Rights—reported as ‘robot judges’ in some of the ensuing media coverage.^148^

While our method of investigating rulings on the (non-)treatment of patients in the MCS has not yielded the predictability of ‘robot judges’, we had hoped that our analysis might similarly enable future litigants to identify the features of cases that point to one outcome as opposed to another (for example, treatment as opposed to non-treatment). Happily, some such indications emerged; less happily, as a more recent ruling indicates, decisions might be even less predictable than the analysis of our cases has suggested. The ruling in *Briggs* was issued after our period of data collection and analysis had concluded, so was not included in our sample.^149^ Our cases implied, *inter alia*, that the judges prefer that a patient with a long life expectancy (continue to) be treated and that medical opinion will outweigh family opinion. If these are trends, then they are called into question by *Briggs*, in which the patient’s life expectancy appeared to be in the region of 9–10 years, and the family’s views—particularly on what the patient would (not) want—proved influential to the court’s decision that treatment cease. Evidently, we should continue to monitor any themes and trends as the pool of cases grows, to ascertain whether the court’s approaches will further settle and, indeed, whether there is an evolution in judicial approach, towards a more patient or family-oriented perspective.

## X. CONCLUSION

The necessary caveats notwithstanding, key features did emerge from the MCS cases we analysed, which begin to reveal the courts’ approaches to these cases. First, the judges appear keen to frame the cases in such a way that these are rightly matters for judicial determination: a tragic, contested, urgent, complex, and unique case is one fit for a judge. Secondly, the judges will appraise the types and forms of expertise that enter the courtroom, and they seem to prefer the ‘objective’ and ‘scientific’, and in particular the views of the doctors involved. Thirdly, the judges appear alert to the reasonableness of the evidence (and, indeed, the parties) and will look favourably on parties who are willing to co-operate. This does not mean, however, that the judges will straightforwardly endorse any consensus reached by the parties; rather, the judges will reach their own decisions. Those decisions must be taken on the basis of the best interests of the patient. Our fourth set of findings reveals different approaches to this assessment: some judges will draw up a balance sheet, while others will not—and even when that approach is taken, different decisions might result, despite the recurring presence of similar factors on the balance sheet. However, consistency of approach and predictability of outcome become more evident in our fifth set of findings, in which we see the courts seeking (scientific) certainty. Certainty in diagnosis—either VS or MCS—will determine the court’s general approach, albeit without entirely indicating the likely outcome, at least for MCS patients. However, the outcome for the latter patients does potentially become more predictable when certain prognostic information is revealed: if the patient’s chances of recovery are low or the patient is close to death, a decision not to treat appears more likely to be issued.

*Briggs*, of course, reminds us that it is early days, and decisions about patients in the MCS still remain scarce. Time will tell whether the themes identified here will persist as more cases come before the courts. For now, it seems there are some cues for future litigants to pick up, such as the courts’ apparent preference for co-operation and their likely reliance on the medical evidence, particularly around diagnosis and prognosis. Whether these are the right cues, which will lead to the ‘right’ decisions remains to be seen. Insofar as there is still evidence of inconsistency and unpredictability—for example, around the deployment of a balancing exercise—this should be a matter for concern. But examining inconsistency will only take us to the realisation that two or more answers are possible *in practice*. Which should be the approach taken, or the answer provided, *in principle* will merit further investigation.

## Footnotes

1 A Bainham, ‘Handicapped Girls and Judicial Parents’ (1987) 103 L Quart Rev 334, 339.

2 Eg R Huxtable, *Law, Ethics and Compromise at the Limits of Life: To Treat or Not to Treat?* (Routledge 2012).

3 S Bunn and Z Fritz, *Vegetative and Minimally Conscious States* (POST Note 489, Parliamentary Office of Science and Technology March 2015), 1.

4 Including the public bodies that fund treatment, which may, on a crude cost basis, be unwilling to initiate legal proceedings, if they feel the outcome of proceedings is uncertain.

5 PJ Koehler and EF Wijdicks, ‘Historical Study of Coma: Looking Back through Medical and Neurological Texts’ (2008) 131 Brain 877–89.

6 Royal College of Physicians (2013) *Prolonged disorders of consciousness: National clinical guidelines*, London: RCP.

7 Koehler and Wijdicks (n 5).

8 As ‘Apallic Syndrome’. See: E Kretschmer, ‘Das Apallische Syndrome’ (1940) 169 Gesamte Neurol Psychiatr 576.

9 B Jennett and F Plum, ‘Persistent Vegetative State after Brain Damage. A Syndrome in Search of a Name’ (1972) 1(7753) Lancet 734.

10 Royal College of Physicians (n 6).

11 JT Giacino and others, ‘Monitoring Rate of Recovery to Predict Outcome in Minimally Responsive Patients’ (1991) 72(11) Arch Phys Med Rehabil 897; DD Tresch and others, ‘Clinical Characteristics of Patients in the Persistent Vegetative State’ (1991) 151(5) Arch Intern Med 930; NL Childs, WN Mercer and HW Childs, ‘Accuracy of Diagnosis of Persistent Vegetative State’ (1993) 43(8) Neurology 1465; K Andrews and others, ‘Misdiagnosis of the Vegetative State: Retrospective Study in a Rehabilitation Unit’ (1996) 313(7048) BMJ 13.

12 JT Giacino, and others, ‘The Minimally Conscious State: Definition and Diagnostic Criteria’ (2002) 58(3) Neurology 349.

13
*In re Quinlan* (1976) NJ 355 A 2d 647. See eg PDG Skegg, ‘The Termination of Life-Support Measures and the Law of Murder’ (1978) 41 Modern Law Review 423.

14
*Airedale NHS Trust v Bland* [1993] AC 789.

15 Department for Constitutional Affairs, *The Mental Capacity Act 2005: Code of Practice* (The Stationery Office, London, 2007), [6.18], [8.18]; Court of Protection Rules, Practice Direction 9E.

16 Eg S Halliday, A Formby and R Cookson, ‘An Assessment of the Court's Role in the Withdrawal of Clinically Assisted Nutrition and Hydration from Patients in the Permanent Vegetative State’ (2015) 23(4) Med L Rev 556; C Kitzinger and J Kitzinger, ‘Court Applications for Withdrawal of Artificial Nutrition and Hydration from Patients in a Permanent Vegetative State: Family Experiences’ (2016) 42(1) J Med Ethics 11.

17
*W v M and S and A NHS Primary Care Trust* [2011] EWHC 2443, per Baker J at [85].

18
*NHS Trust A v H* [2002] 1 FCR 713.

19 The cases we know of are: *Frenchay Healthcare NHS Trust v S* [1994] 2 All ER 403; *Re D* [1997] 38 BMLR 1; *Re H (adult: medical treatment)* [1998] 3 FCR 174.

20
*W v M* (n 17).

21 Court of Protection Rules, Practice Direction 9E, [5].

22 s 2(1).

23 s 1(2).

24 s 3(1).

25 ss 24–26, 9–14, respectively.

26 s 4(6).

27 ‘Best Interests in Medical Ethics and Law (BABEL)’, initially funded by a Wellcome Trust Seed award (110076/Z/15/Z).

28 Such transparency is increasingly called for by funders anxious to understand how research is conducted.

29 Provision of CANH is at issue in 12 of the 17 judgments examined.

30 No date limits were imposed. Cases were included if they concerned decision(s) about (non-)treatment in the best interests of a person who was, or possibly was, in a MCS.

31 As we were finalising the manuscript, an eighteenth case was being decided: *Briggs v Briggs* [2016] EWCOP 53. In a ground-breaking development, permission was granted for live tweeting of the proceedings by @KitzingerCelia, @JennyKitzinger and@cdocuk using #COPBriggs.

32 V Braun and V Clarke, ‘Using Thematic Analysis in Psychology’ (2006) 3(2) Qual Res Psychol 77.

33 J Thomas and A Harden, ‘Methods for the Thematic Synthesis of Qualitative Research in Systematic Reviews’ (2008) 8 BMC Med Res Methodol 45.

34 Eg SJ Ellis and C Kitzinger, ‘Denying Equality: An Analysis of Arguments against Lowering the Age of Consent for Sex between Men’ (2002) 12(3) J Comm & Applied Social Psychol 167. cf RJ Cahill-O’Callaghan, ‘Reframing the Judicial Diversity Debate: Personal Values and Tacit Diversity’ (2015) 35(1) LS 1 (note that Cahill-O’Callaghan used a *deductive* process, in which a set of codes were imposed on the data, which differs from the *inductive* process of coding and theme formation which we undertook).

35 Above (n 32).

36 Ellis and Kitzinger (n 34).

37 The remaining codes and themes, many of which relate to ethical values in the judgments, will be addressed in a separate article.

38 Eg *Re H* (n 19), per Sir Stephen Brown P at 175.

39 Eg *An NHS Trust v L* [2013] EWHC 4313, per Moylan J at [102].

40
*County Durham & Darlington NHS Foundation v PP, H, D and S* [2014] EWCOP 9, per Cobb J at [8].

41
*St George’s v P* [2015] EWCOP 42, per Newton J at [7].

42
*A v H* (n 18).

43 ibid.

44
*W v M* (n 17).

45
*Re S* [2016] EWCOP 32, per Hayden J at [13].

46
*Frenchay* (n 19).

47 Eg *United Lincolnshire Hospitals NHS Trust v N* [2014] EWCOP 16; *Re D* (n 19).

48 We should note, however, that some sort of distinction *might* hold in some cases, in which a narrow ‘window of opportunity’ for non-treatment is perceived: see J Kitzinger and C Kitzinger, ‘The “window of opportunity” for Death after Severe Brain Injury: Family Experiences’ (2013) 35(7) Sociol Health and Illness 1095.

49 Besides *NHS v VT and A* [2014] COPLR 44, the instances are: *St George’s v P* (n 41), per Newton J at [18]; *Aintree University Hospitals NHS Foundation Trust v James* [2013] UKSC 67, per Lady Hale at [36]; and *W v M* (n 17) at [75].

50
*NHS v VT*, ibid per Hayden J at [20].

51
*Portsmouth NHS Trust v Wyatt* [2004] EWHC 2247, at [23].

52 See n 16.

53 Eg Mental Capacity Act 2005, s 4(6).

54 Eg *W Healthcare Trust v KH, H and PH* [2004] EWCA 1324; *W v M* (n 17).

55 Eg *Re D* (n 19); *M v N* [2015] EWCOP 76.

56
*A v H* (n 18), per Dame Butler-Sloss P at 718.

57
*St George’s v P* (n 41), per Newton J at [40].

58
*Frenchay* (n 19), per Sir Thomas Bingham MR at 405.

59 Eg noting that an expert witness in *VT* (n 49) chaired a clinical ethics committee (at [9]).

60 The judge imputes ethical sensitivity and ‘compassion’ to a medical witness in *Lincolnshire* (n 47), per Pauffley J at [62–63].

61
*M v N* (n 55), per Hayden J at [33–34].

62 ibid at [22].

63 In our series, this happens once: *St George’s v P* (n 41).

64 Three cases affirm this principle: *Frenchay* (n 19); *L* (n 39), at [116]; *VT* (n 49) at [27], with a fourth case, *St George’s v P* (n 41) seeming to contradict this principle: ‘There is almost nothing to rebut the very strong presumption that it is in P's best interests to stay alive. I order and direct that the renal replacement therapy should continue’ (per Newton J at [45]). For further discussion of the implications of this judgment see: JC Youngs, ‘Can the Courts Force the Doctor's Hand? *St George's Healthcare NHS Trust v P* [2015] EWCOP 42’ (2016) 24 Med L Review 99.

65
*Re J (a minor) (medical treatment)* [1992] 2 FCR 753, per Lord Donaldson MR at 762.

66
*VT* (n 49), per Hayden J at [27].

67
*W v M* (n 17), per Baker J at [251].

68
*Lincolnshire* (n 47), per Pauffley J at [58].

69
*Re H* (n 19), per Sir Stephen Brown P at 177.

70
*Re JD* [2012] EWHC 4420, per Roderic Wood J at [22].

71
*W v M* (n 17), at [258].

72
*Re S* (n 45), per Hayden J at [13].

73
*M v N* (n 55), per Hayden J at [22].

74
*W v M* (n 17), per Baker J at [254].

75 Eg *Sheffield Teaching Hospitals NHS Foundation Trust v TH and another* [2014] EWCOP 4, per Hayden J at [5].

76 Eg *Re J (A Minor) (Wardship: Medical Treatment)* [1991] Fam 33, at 41. For commentary on shared decision-making as an ethical concept see: L Sandman and C Munthe, ‘Shared Decision Making, Paternalism and Patient Choice’ (2010) 18 Health Care Anal 60.

77
*L* (n 39) at [31].

78 Above (n 15) at [5.31].

79 s 4(6).

80
*Aintree* (n 49), per Lady Hale at [45].

81
*Frenchay* (n 19) at 411.

82 Eg *Re JD* (n 70), per Roderic Wood J at [9].

83 Eg *KH* (n 54); *L* (n 39).

84
*M v N* (n 55), per Hayden J at [63]. In our case series, such an occurrence is less rare than Hayden J indicates here eg *Re JD* (n 70); *PP* (n 40); *Gloucestershire Clinical Commissioning Group v AB and another* [2014] EWCOP 49.

85
*An NHS Trust v DJ and others* [2012] EWHC 3524.

86
*Aintree* (n 49), per Lady Hale at [12].

87
*VT* (n 49).

88 ibid at [27–28]. Hayden’s strategy of separating the analysis is founded on *obiter* statements by Lady Hale in *Aintree* (n 49).

89 Huxtable (n 2).

90 Although not cited in this case, the solution (and sense of tentative compromise) is very similar to the approach taken by Theis J in *An NHS Trust v AB* [2014] EWHC 1031, a case on which we have commented in G Birchley and R Huxtable, ‘Critical Decisions for Critically Ill Infants: Principles, Processes, Problems’ in C Stanton and others (eds), *Pioneering Healthcare Law: Essays in Honour of the Work of Margaret Brazier* (Routledge 2015) 116.

91
*VT* (n 49), per Hayden J at [28].

92
*Re A (Male Sterilisation)* [2000] 1 FCR 193, per Thorpe LJ at 203.

93 Mr Justice Baker, ‘A Matter of Life and Death’ Oxford Shrieval Lecture, 11 October 2016 <https://www.judiciary.gov.uk/wp-content/uploads/2016/10/mr-justice-baker-shrieval-lecture-11102016.pdf> accessed 16 December 2016 (emphasis added).

94
*Frenchay* (n 19); *Re D* (n 19); *Re H* (n 19); *A v H* (n 18); *Re JD* (n 70); *Gloucestershire* (n 84); *Re S* (n 45).

95
*Sheffield* (n 75).

96
*PP* (n 40). Since PP’s awareness was found to be between VS and the lower margin of MCS, it is probable that she was treated as a PVS patient by the judge.

97
*VT* (n 49) [20].

98
*M v N* (n 55) at [70].

99 In appeal cases, the balance sheet provided at first instance stands, as it is not in the ambit of the higher court to overturn the balancing exercise of the lower: see *KH* (n 54), per Brooke LJ at [28].

100
*KH* (n 54).

101
*W v M* (n 17) at [248].

102 ibid at [247].

103
*L* (n 39) at [121].

104 ibid at [122]

105
*DJ* (n 85). Repeated in *Aintree* (n 49) at [11].

106 ibid, per Jackson J at [79].

107 ibid.

108
*Lincolnshire* (n 47) at [59].

109 ibid at [58].

110
*St George’s v P* (n 41) at [43]; see also [35]–[38].

111 ibid.

112 Above (n 93). He cites *Re F (A Child) (International Relocation Cases)* [2015] EWCA Civ 882.

113
*A v H* (n 18), per Dame Butler-Sloss P at 717.

114
*W v M* (n 17), per Baker J at [55].

115 See Section II.

116 These changing approaches are mirrored by changing perceptions of families whose relationships with their relative and the carers is also dynamic. Thus, families lose faith in carers and hospitals (eg *St George’s v P* (n 41) at [7]) or question their own motivations for wishing to keep their relative alive (eg *W v M* (n 18), at [15]).

117 Eg *Aintree* (n 49), per Lady Hale at [6]; *An NHS Trust v L* (n 39), per Moylan J at [103] and *passim*.

118 In contrast with the position on VS patients. In *Bland*, Lord Keith noted that court approval was needed ‘at least for the time being and until a body of experience and practice has been built up’ (above n 14, at 859).

119
*St George’s v P* (n 41), per Newton J at [13].

120 Royal College of Physicians (n 6).

121 Eg *Re S* (n 45), per Hayden J at [8–9]. Other examples of uncertainty include: *Gloucestershire* (n 84); *Aintree* (n 49), per Lady Hale at [41].

122 Eg *Re JD* (n 70); *Re S* (n 45).

123 See *An NHS Trust v J* [2006] All ER (D) 73 (Dec).

124
*PP* (n 40), per Cobb J at [33].

125
*PP* (n 40), per Cobb J at [11].

126
*L* (n 39), per Moylan J at [25].

127 We say ‘might’, as it is not absolutely clear that prognosis is determinative. Such prognoses are open to dispute (as is the case in *Sheffield* (n 75)), a problem that might undermine their ability to influence the judgment.

128
*W v M* (n 17), per Baker J at [203].

129
*Frenchay* (n 19), per Sir Thomas Bingham MR at 406 (emphasis added).

130
*M v N* (n 55) at [74].

131
*St George’s v P* (n 41), at [37].

132
*W v M* (n 17), per Baker J at [26].

133
*Sheffield* (n 75), per Hayden J at [24].

134 Such reasoning reflects the approach taken in the seminal American decision in *Quinlan*, in which the court indicated that the governmental interest in the preservation of life weakens ‘as the prognosis dims’: *Quinlan*, (n 13), per Hughes CJ at 41.

135 Eg C Kitzinger and J Kitzinger, ‘Withdrawing Artificial Nutrition and Hydration from Minimally Conscious and Vegetative Patients: Family Perspectives’ (2015) 41(2) J Med Eth 157.

136 Eg A Mullock, ‘Deciding the Fate of a Minimally Conscious Patient: An Unsatisfactory Balancing Act?: *W v M And Others* [2011] EWHC 2443 (Fam)’ (2012) 20(3) Med LR 460; D Wilkinson and J Savulescu, ‘Is it Better to be Minimally Conscious than Vegetative?’ (2013) 39(9) J Med Eth 557; R Huxtable, ‘“In a Twilight World”: Judging the Value of Life for the Minimally Conscious Patient” (2013) 39(9) J Med Eth 565; R Heywood, ‘Moving on from *Bland*: The Evolution of the Law and Minimally Conscious Patients’ (2014) 22(4) Med LR 548; R Huxtable, ‘From Twilight to Breaking Dawn? Best Interests, Autonomy, and Minimally Conscious Patients: *M v N* [2015] EWCOP 76 (Fam)’ (2016) Med LR doi: 10.1093/medlaw/fww008.

137
*Bolam v Friern Hospital Management Committee* [1957] 2 All ER 118. See eg M Brazier and J Miola, ‘Bye-Bye Bolam: A Medical Litigation Revolution?’ (2000) 8 Med LR 85.

138
*Bolitho (Deceased) v City and Hackney HA* [1998] AC 232; *Montgomery v Lanarkshire* [2015] UKSC 11.

139 The first author is grateful to Clark Hobson for his insightful observations on this point.

140 C Kitzinger and J Kitzinger, ‘Withdrawing Artificial Nutrition and Hydration from Minimally Conscious and Vegetative Patients: Family Perspectives’ (2015) 41(2) J Med Ethics 157.

141 N Aletras, and others, ‘Predicting Judicial Decisions of the European Court of Human Rights: A Natural Language Processing perspective’ (2016) 2 Peer J Comput Sci e93.

142 Which we seek to undertake in a future article.

143 J Harrington, ‘Deciding Best Interests: Medical Progress, Clinical Judgment and the ‘Good Family’ (2003) 3 Web JCLI.

144 P Birks, ‘Rights, Wrongs, and Remedies’ (2000) 20(1) Oxford Journal of Legal Studies 1, 2–3.

145 L Fuller, *The Morality of Law* (revised edn, Yale University Press 1969) 96.

146 E.g. J Raz, ‘The Rule of Law and its Virtue’ (1977) 93 The Law Quarterly Review 195; J Waldron, ‘The Rule of Law in Contemporary Liberal Theory’ (1989) 2(1) Ratio Juris 79, 84.

147 Aletras (n 141).

148 A Griffin, ‘Robot Judges could Soon be Helping with Court Cases’ *The Independent* (London, 24 October 2016) <http://www.independent.co.uk/life-style/gadgets-and-tech/news/ai-judge-robot-european-court-of-human-rights-law-verdicts-artificial-intelligence-a7377351.html> accessed 16 December 2016.

149
*Briggs* (n 31).

